# Brain metabolism response to intrahospital transfers in neurocritical ill patients and the impact of microdialysis probe location

**DOI:** 10.1038/s41598-024-57217-5

**Published:** 2024-03-28

**Authors:** Leire Pedrosa, Jhon Hoyos, Luis Reyes, Alejandra Mosteiro, Luigi Zattera, Thomaz Topczewski, Ana Rodríguez-Hernández, Sergio Amaro, Ramon Torné, Joaquim Enseñat

**Affiliations:** 1 Department of Neurosurgery, Hospital Clinic of Barcelona, 08036 Barcelona, Spain; 2 IDIBAPS Biomedical Research Institute, 08036 Barcelona, Spain; 3 Department of Anesthesiology and Critical Care, Hospital Clinic of Barcelona, 08036 Barcelona, Spain; 4 Department of Neurosurgery, Germans Trias i Pujol University Hospital, 08916 Badalona, Spain; 5 Comprehensive Stroke Unit, Neurology, Hospital Clinic of Barcelona, 08036 Barcelona, Spain

**Keywords:** Microdialysis monitoring, Brain metabolism, Intrahospital transfer, Neurocritical patients, Traumatic brain injury, Subarachnoid hemorrhage, Neuroscience, Biomarkers, Medical research, Neurology

## Abstract

Intrahospital transfer (IHT), a routine in the management of neurocritical patients requiring imaging or interventions, might affect brain metabolism. Studies about IHT effects using microdialysis (MD) have produced conflicting results. In these studies, only the most damaged hemisphere was monitored, and those may not reflect the impact of IHT on overall brain metabolism, nor do they address differences between the hemispheres. Herein we aimed to quantify the effect of IHT on brain metabolism by monitoring both hemispheres with bilateral MD. In this study, 27 patients with severe brain injury (10 traumatic brain injury and 17 subarachnoid hemorrhage patients) were included, with a total of 67 IHT. Glucose, glycerol, pyruvate and lactate were measured by MD in both hemispheres for 10 h pre- and post-IHT. Alterations in metabolite levels after IHT were observed on both hemispheres; although these changes were more marked in hemisphere *A* (most damaged) than *B* (less damaged). Our results suggest that brain metabolism is altered after an IHT of neurocritical ill patients particularly but not limited to the damaged hemisphere. Bilateral monitorization may be more sensitive than unilateral monitorization for detecting metabolic disturbances not directly related to the course of the disease.

## Introduction

Intrahospital transfer (IHT) is a routine process in intensive care units (ICU) for neurocritical patients^1^. Actually, at least two IHTs are needed when an advanced imaging study or surgical or interventional treatments are deemed necessary. Most centers try to minimize the number of IHTs of neurocritical patients, as they may result in airway management problems, medication disruption, positional changes, and ventricular drainage discontinuation^2–5^. These situations experienced during IHTs may cause an increase in intracranial pressure (ICP) and/or brain hypoxia leading to secondary brain injury (SBI), thus worsening the clinical outcome.

Metabolic dysregulations may reflect this iatrogenic SBI and therefore cerebral microdialysis (MD) monitoring has been one of the tools used to investigate the consequences of IHT in neurocritical patients. However, the results obtained so far are diverse and contradictory, ranging from the absence of metabolite changes during or after IHT to increases in glycerol, lactate and pyruvate levels immediately after IHT^6^.

In these previous studies, the MD monitorization was conducted unilaterally, specifically in the hemisphere defined as the one most damaged. Nevertheless, the difficulty in defining the hemisphere at higher risk is well known^7–10^ and relevant information may be overlooked when only one hemisphere is monitored^11^. The purpose of our study was to quantify the effect of IHT on brain metabolism of neurocritical patients monitored with bilateral MD probes and to assess the differential impact of the underlying pathology on the presence of bilateral brain metabolism.

## Results

**Figure 1:**
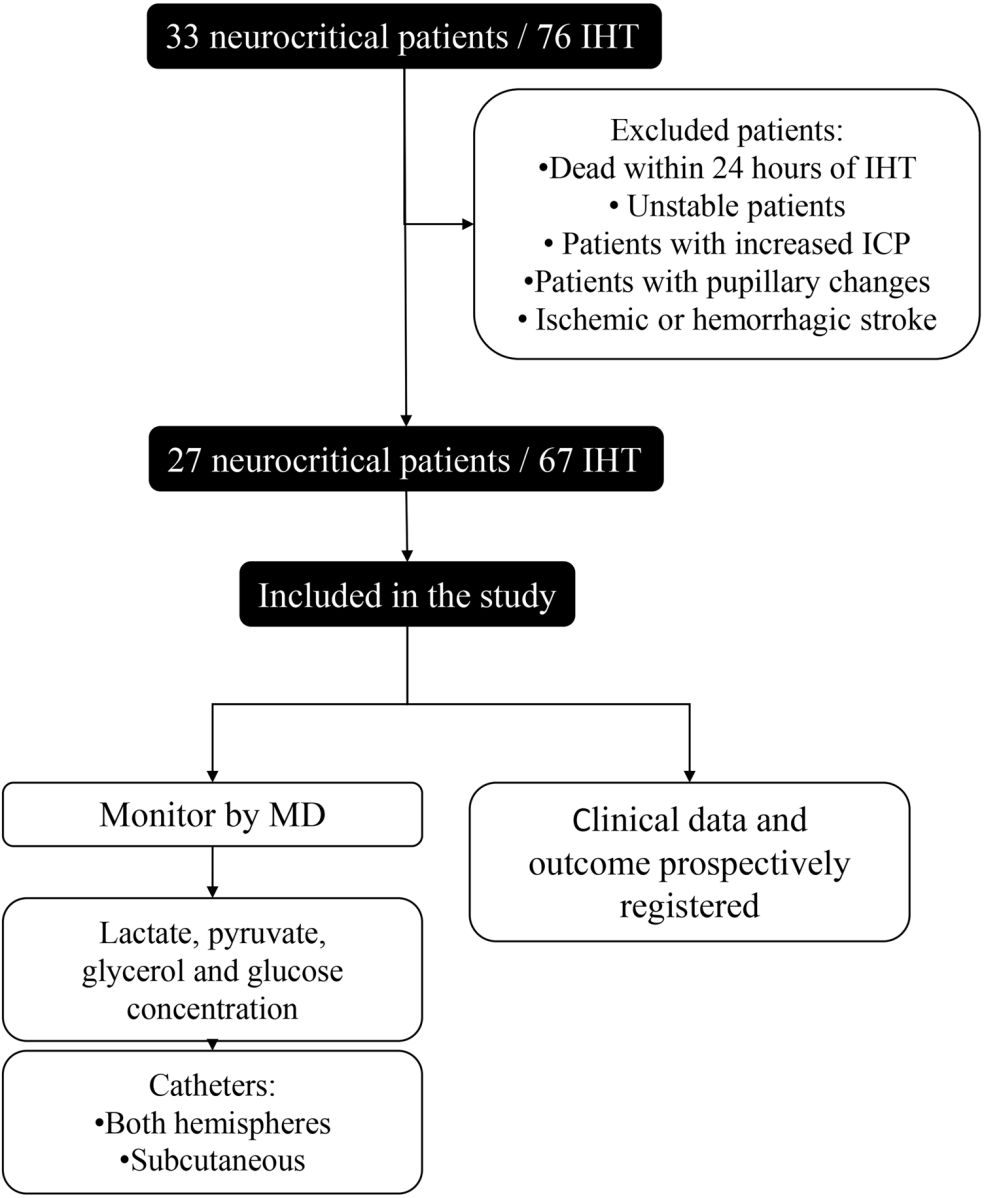
Study flowchart. Initially, 33 neurocritical patients were included in the study. Patient which dead within 24 h post-first IHT, unstable patients, and ischemic or hemorrhagic stroke were excluded. 27 patients were maintained in the study, with a total of 67 IHT, and were monitored by MD. Glucose, glycerol, lactate and pyruvate were analyzed from both cerebral hemispheres. Clinical data of 27 patients were recollected. *IHT* intrahospital transfer, *MD* microdialysis.

Overall, 71 IHT from 29 patients were collected. Three IHT corresponding to two patients who died within 24 h of IHT were excluded from the final analysis. Of the remaining 67 IHT analyzed, the mean transfer time was 21 ± 6 min. A total of 88.06% (59/67) of IHT were done to obtain a CT, 10.44% (7/67) to acquire an angiography, and 1.50% (1/67) to perform an endovascular treatment (Fig. 1).

### Patient characteristics and transfers

Out of the 27 patients analyzed, 17 (17/27; 52%) were male. The mean age was 47 years (range 20–72) and the mean Glasgow Coma Scale (GCS) at admission was 7 (range 3–14). Most of the patients (19/27; 70%) were admitted in coma (GCS ≤ 8). The cause of the brain injury was subarachnoid hemorrhage (SAH) pathology in 17/27 cases (63%) and traumatic brain injury (TBI) in 10/27 (37%). Supplementary Tables S1 and S2 display the information about the injury side, the aneurism location and the surgery side.

Out of the 27 patients, 16 (59%) required surgical intervention, 1 (4%) required a surgical evacuation of an intraparenchymal hematoma, 3 (11%) underwent a decompressive craniectomy, 4 (15%) needed a craniotomy for aneurysm clipping, and 3 (11%) more required aneurysms clipping plus hematoma drainage (Table 1). Only 3 of 17 (17.65%) SAH patients developed delayed cerebral ischemia (DCI). All three patients were treated with vasoactive drugs, and one patient received rescue therapy with balloon angioplasty and intra-arterial verapamil. Overall, good clinical outcome was observed in 36% (9/25) of the patients at hospital discharge and in 74% (14/19) at 3 months follow-up.

**Table 1:** Demographic and clinical variables of the patients included in the study.

		Total (n = 27)	TBI (n = 10)	SAH (n = 17)	p-value
		n (%)	n (%)	n (%)	
Sex	Male	14 (51.85%)	8 (80.00%)	6 (35.30%)	0.065
Age	Mean (min–max)	47 (20–72)	32.3 (20–61)	56.00 (48–72)	** < 0.001**
GCS at hospital admission	Severe (3–8)	19 (70.37%)	8 (80.00%)	11 (64.71%)	0.654
	Moderate (9–12)	5 (18.52%)	1 (10.00%)	4 (23.53%)	
	Good (13–15)	3 (11.11%)	1 (10.00%)	2 (11.76%)	
Surgery	Non-surgery	16 (59.26%)	6 (60.00%)	10 (58.82%)	**0.027**
	Hematoma drainage	1 (3.70%)	1 (10.00%)	0 (0.00%)	
	Aneurysm clipping plus hematoma drainage	3 (11.11%)	0 (0.00%)	3 (17.65%)	
	Decompressive craniectomy	3 (11.11%)	3 (30.00%)	0 (0.00%)	
	Aneurysm clipping	4 (14.82%)	0 (0.00%)	4 (23.53%)	
DCI	Non-DCI	7 (70.00%)	0 (0.00%)	7 (70.00%)	
	Clinical	1 (10.00%)	0 (0.00%)	1 (10.00%)	
	Radiological	2 (20.00%)	0 (0.00%)	2 (20.00%)	
Number of IHT	1	6 (22.22%)	3 (30.00%)	3 (17.65%)	0.243
	2	10 (37.03%)	5 (50.00%)	5 (29.41%)	
	3 or more	11 (40.74%)	2 (20.00%)	9 (52,94%)	
Status on the day of discharge	Bad	16 (64.00%)	4 (40.00%)	12 (80.00%)	0.106
	Good	9 (36.00%)	6 (60.00%)	3 (20.00%)	
Outcome at 3 months	Bad	5 (26.31%)	1 (14.29%)	4 (33.33%)	0.712
	Good	14 (73.69%)	6 (85.71%)	8 (66.67%)	

Data are first described in the global group and then categorized according to the pathological admission diagnosis (SAH or TBI). The table shows the p-value obtained by comparing the subjects in each variable in each pathological group with the chi-square test.

*SAH* subaracnhoid hemorraghe, *TBI* traumatic brain injury, *GCS* glasgow coma scale (GCS), *mRS* modified Rankin scale score, *IHT* intrahospital transfer, *DCI* delayed cerebral ischemia.

Significant values are in bold.

Most of the included patients (11/27; 41%) underwent 3 or more transfers, 10 (10/27; 37%) patients had 2 transfers, and 6 patients (6/27; 22%) had only one transfer. Those patients who required a higher number of transfers were predominantly SAH sufferers (9 SAH vs. 2 TBI patients). The most frequent number of transfers in TBI patients was 2 (5/10; 50%), whereas most SAH patients (9/17; 53%) required 3 or more transfers (Table 1).

### Brain metabolism

**Figure 2:**
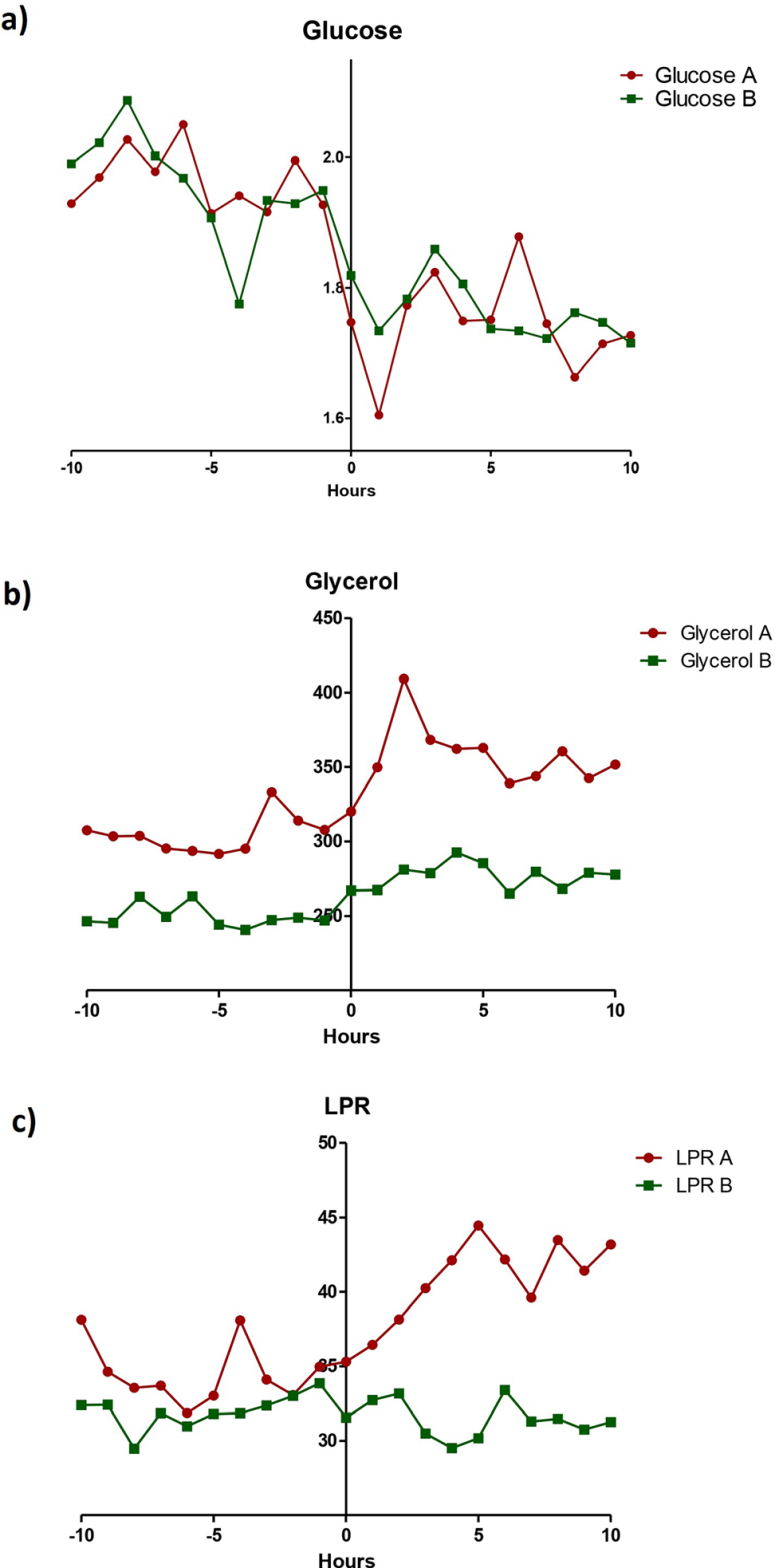
Biochemistry metabolites from both cerebral hemispheres analyzed by microdialysis. Hourly values of cerebral glucose (**a**), cerebral glycerol (**b**), and cerebral lactate/pyruvate ratio (**c**), 10 h before and after the intrahospital transfer of both hemispheres. The interstitial liquid was analyzed from the affected hemisphere (A, red) and non-affected hemisphere (B, green). *LPR* lactate/pyruvate ratio.

Before IHT, the levels of glycerol and lactate/piruvate ratio (LPR) were higher on *A side* than on *B side* (mean 304.60 vs 249.60 µmol/l, and 34.52 vs 32.00, respectively), whereas the levels of glucose (1.96 vs 1.96 mmol/l) were similar in both hemispheres (p > 0.05). After IHT, our data showed a decrease in glucose (1.96 mmol/l pre-IHT vs 1.74 mmol/l post-IHT, p < 0.05), an increase in glycerol (304.60 µmol/l pre-IHT vs 358.90 µmol/l post-IHT, p < 0.05) and an increase in LPR (34.50 pre-IHT vs 41.13 post-IHT, p < 0.05) on *A side*. Similarly, we also observed a decrease in glucose (1.96 mmol/l pre-IHT vs 1.76 mmol/l post-IHT, p < 0.05), and a significant increase in glycerol (245 µmol/l pre-IHT vs 272 µmol/l post-IHT, p < 0.05) on *B side*. In contrast to *A side*, on *B side* no statistically significant differences in LPR were observed (32.00 pre-IHT vs 31.43 post-IHT, p = 0.935) (Fig. 2 and Table 2). In addition, a statistically significant increase in pyruvate was observed on *A side*, while this difference was absent on *B side*.

**Table 2:** Metabolites analyzed by MD in both hemispheres (*A* and *B side*) and in control side *(C side)* pre- and post-IHT.

	A side			B side			C side		
	Pre IHT	Post IHT	p-valor	Pre IHT	Post IHT	p-valor	Pre IHT	Post IHT	p-valor
Glucose (mmol/L) ± SD	1.96 ± 0.94	1.74 ± 0.91	**0.0002**	1.96 ± 1.43	1.76 ± 1.19	**0.0334**	2.66 ± 1.36	2.66 ± 2.36	0.1528
Glycerol (Umol/L) ± SD	304.60 ± 312.0	358.90 ± 330.10	**0.0019**	249.60 ± 214.10	277.50 ± 196.0	**0.00933**	215.50 ± 93.85	216.90 ± 103.50	0.802
Lactate (mmol/L) ± SD	3.99 ± 3.02	4.69 ± 3.23	** < 0.0001**	3.84 ± 3.21	3.84 ± 2.79	**0.0345**	1.22 ± 0.83	1.19 ± 0.80	0.8442
Pyruvate (Umol/L) ± SD	123.90 ± 68.25	133.60 ± 68.25	**0.0012**	112.50 ± 57.75	119.20 ± 56.88	0.1156	64.47 ± 34.98	57.54 ± 35.99	0.3574
LPR ± SD	34.52 ± 28.58	41.13 ± 47.81	**0.0329**	32.00 ± 16.56	31.43 ± 13.40	0.9353	21.30 ± 11.08	25.05 ± 18.35	0.2659

Mean concentration of each metabolite and SD from affected (*A side*) and non-affected (*B side*) hemispheres, and control side *(C side*) were shown. The p-value was obtained by comparing the values pre and post-IHT with the *t* test student.

*IHT* intrahospital transfer, *LPR* lactate/pyruvate ratio.

Significant values are in bold.

In order to monitor systemic levels of these metabolites, a subcutaneous catheter (C side) was inserted. No statistically significant differences were observed in any of the studied metabolites (glucose, glycerol, pyruvate, lactate) before and after IHT on the C side.

**Figure 3:**
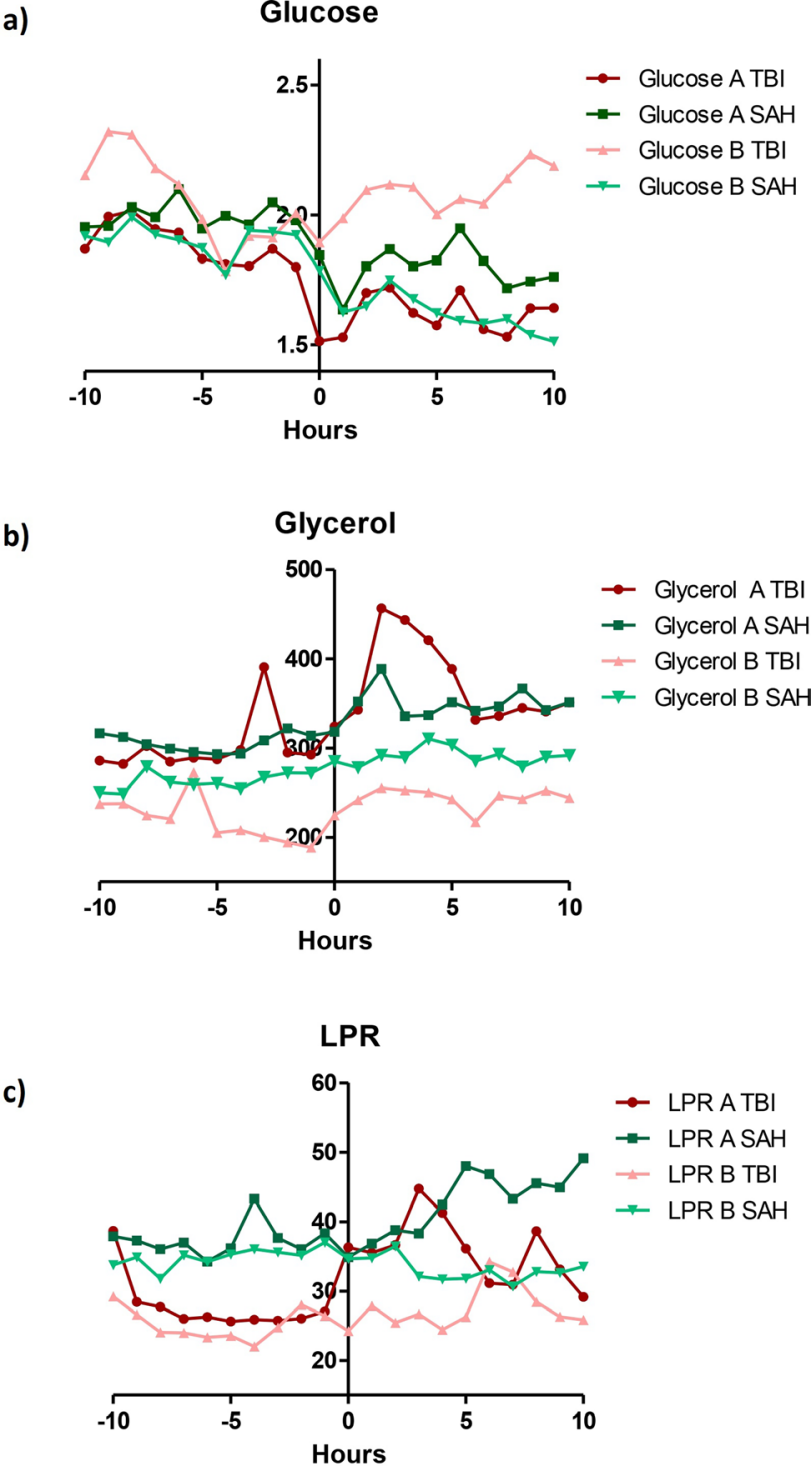
Biochemistry metabolites from both cerebral hemispheres analyzed by microdialysis of TBI and SAH patients. Hourly values of cerebral glucose (**a**), cerebral glycerol (**b**), and cerebral lactate/pyruvate ratio (**c**), 10 h before and after the intrahospital transfer, comparing both hemispheres (affected hemisphere (A; red and green) and non-affected hemisphere (B; turquoise and pink) of TBI and SAH patients. *SAH* subarachnoid hemorrhage, *TBI* traumatic brain injuty, *LPR* lactate/pyruvate ratio.

Regarding the results obtained in the most affected side (*A side*) according to the underlying pathology (SAH vs TBI), we found a decreased level of glucose before and after IHT in both pathologies: TBI patients (1.89 mmol/l pre-IHT vs 1.62 mmol/l post-IHT, p = 0.016) and SAH patients (2.00 mmol/l pre-IHT vs 1.80 mmol/l post-IHT, p = 0.008). Besides, there was also an increase in LPR in TBI patients (28 pre-IHT vs 36 post-IHT, p = 0.055) and in the SAH pathology group (32.78 pre-IHT vs 35.87 post-IHT, p = 0.22) (Fig. 3 and Table 3).

**Table 3:** Metabolites analyzed by MD in both hemispheres (*A* and *B side*) and in control side *(C side)* pre- and post-IHT.

Metabolite	Pathology	A side			B side			C side		
		Pre IHT	Post IHT	p-value	Pre IHT	Post IHT	p-value	Pre IHT	Post IHT	p-value
Glucose (mmol/L) ± SD	TBI	1.888 ± 1.048	1.624 ± 0.9814	**0.016**	2.069 ± 0.9855	2.099 ± 1.427	0.225	3.71 ± 1.57	4.69 ± 4.23	0.8125
	SAH	2.00 ± 0.90	1.80 ± 0.90	**0.0084**	1.89 ± 1.60	1.57 ± 1.03	**0.035**	2.38 ± 1.183	2.11 ± 1.88	0.126
Glycerol (Umol/L) ± SD	TBI	300.9 ± 380.2	376.0 ± 400.3	**0.012**	219.1 ± 266.7	244.8 ± 219.5	0.059	277.70 ± 136.70	296.60 ± 138.80	0.6194
	SAH	306.1 ± 282.8	351.70 ± 299.90	**0.033**	259.30 ± 190.10	289.30 ± 187.30	**0.049**	198.80 ± 73.57	195.40 ± 82.65	0.8192
Lactate (mmol/L) ± SD	TBI	3.470 ± 3.642	4.305 ± 3.904	**0.001**	2.366 ± 1.651	2.378 ± 1.109	0.059	1.55 ± 0.93	1.47 ± 0.94	0.6875
	SAH	4.22 ± 2.70	4.851 ± 2.926	**0.0008**	4.46 ± 3.50	4.46 ± 3.06	0.1641	1.13 ± 0.80	1.11 ± 0.77	0.9393
Pyruvate (Umol/L) ± SD	TBI	116.8 ± 79.73	123.2 ± 81.02	0.185	92.25 ± 44.06	91.77 ± 31.08	0.255	78.23 ± 37.66	72.58 ± 46.87	0.7127
	SAH	126.90 ± 63.46	138.00 ± 58.92	**0.0029**	121.20 ± 61.06	130.80 ± 61.47	0.0149	60.76 ± 34.03	53.50 ± 32.40	0.226
LPR ± SD	TBI	27.75 ± 12.94	35.73 ± 23.00	0.055	25.20 ± 9.927	27.83 ± 9.064	0.253	20.70 ± 10.19	21.47 ± 11.29	0.5781
	SAH	32.78 ± 12.38	35.87 ± 17.52	0.229	34.90 ± 18.00	32.96 ± 14.68	0.446	21.47 ± 11.52	26.02 ± 19.90	0.2818

Mean concentration of each metabolite and SD from affected (*A side*) and non-affected (*B side*) hemispheres, and control side *(C side*) were shown. Patients were grouped according to the pathological diagnosis (SAH and TBI). The p-value was obtained by comparing the values pre- and post-IHT with the *t* test student.

*SAH* subarachnoid hemorrhage, *TBI* traumatic brain injury, *IHT* intrahospital transfer, *LPR* lactate/pyruvate ratio.

Significant values are in bold.

Meanwhile, in *B side,* a number of differences were observed between pre- and post-IHT periods in the SAH pathology group including increases in glycerol levels (259.30 µmol/l pre-IHT vs 289.30 µmol/l post-IHT, p = 0.049) and pyruvate levels (121.20 µmol/l pre-IHT vs 130.80 µmol/l post-IHT, p = 0.015), and decreases in glucose levels (1.89 mmol/l pre-IHT vs. 2.57 mmol/l post-IHT, p = 0.035). Conversely, no statistically significant differences were found in the LPR (34.90 pre-IHT vs 32.96 post-IHT, p = 0.446) nor in lactate levels (4.46 mmol/l pre-IHT vs 4.46 mmol/l post-IHT, p = 0.164). Interestingly, in SAH patients, the LPR on *B side* did not change after the transfer, as compared to the increase observed on *A side*. In the TBI group, no differences were seen in the metabolic parameters on *B side* before and after IHT (Fig. 3 and Table 3).

Comparing the metabolite values in SAH patients who did not developed DCI, we also observed the same significant changes, suggesting that the observed changes were not due to the presence of DCI, but rather to the IHT (Supplementary Table S3).

Additionally, the levels of all metabolites on C side remain constant before and after IHT. There are no significant differences observed in the levels of glucose, lactate, pyruvate, or LPR before and after IHT.

## Discussion

The clinical repercussions of IHT in neurocritical patients have been debated over the last years^1,2^, with some authors advocating keeping the patient in intensive care unit (ICU) as much as possible thus reducing transfer outside the unit. However, balancing the potential risk and benefits of IHT is not an easy task. On one hand, diagnostic tests and therapeutic interventions requiring an IHT may be lifesaving. On the other hand, the metabolic response of the brain to IHT may cause SBI thus hindering the odds of patient recovery. However, the brain response to IHT is not well understood, especially regarding the less-affected hemisphere, which is usually not monitored. Furthermore, data on whether this response may vary depending on the underlying pathology is also lacking. In this scenario, the present study aimed to assess the impact of IHT on brain metabolism in neurocritical patients through the use of bilateral MD monitoring. Interestingly, our results suggest that IHT affects the brain metabolism not only in the hemisphere with more damage (*A side*), but it also on the side with less initial injury (*B side*). Moreover, we observed that this *B side* impairment seems to vary according to the underlying pathology. Therefore, this “contralateral” response to IHT might be significant in the patient’s overall prognosis and could be of greater importance in pathologies with more diffuse damage.

Our findings revealed significant effects of IHT on cerebral metabolism, particularly on the more damaged side (A). Specifically, on *A side* we observed after IHT a notable increase in glycerol levels, a significant decrease in glucose levels, and a significant increase in the LPR. These changes may be attributed to episodes of hypermetabolism or “metabolic crises”, which have been linked to unfavorable outcomes^12–14^. The stress induced by IHT could be responsible for the observed decrease in glucose levels though the enhancement of anaerobic glycolysis and eventually resulting in an elevated LPR^15^. Importantly, an elevated LPR has been associated with the development of delayed cerebral ischemia (DCI) in SAH patients, suggesting that IHT may increase the risk of DCI following transfers^16^.

In previous studies^6,8–10,17,18^, similar results were obtained regarding the increase in glycerol after IHT. However, in contrast with our observed data some of these studies did not find variations in other parameters of brain metabolism. Küchler et al. found no difference in metabolite levels before and after IHT in intracranial hemorrhage (ICH), SAH and brain trauma. The study concluded that although most of the metabolites analyzed increased after IHT, these changes were not related to incident metabolic crisis^18^. Contrarily and in line with our results, Hosman et al. found significant increases in difference glycerol, lactate and pyruvate levels after IHT, suggesting that IHT affects the brain metabolism of neurocritical patients^6^.

The main novelty provided by our investigation was the assessment of the effects of IHT on cerebral metabolism in patients monitored with bilateral MD. Similar to the metabolite changes found on *A side*, we also observed on *B side* a significant increase in post-transfer glycerol levels, indicating acute neuronal damage. However, while glucose levels decreased significantly after transfer on *B side*, no significant changes in the LPR were observed. This suggests that *B side* did not experience the same level of stress-induced anaerobic glycolysis as that observed on *A side.* Furthermore, analyzing metabolism disturbances by pathology, we observed that TBI patients presented a significant decrease in glucose, an increase in glycerol and an increase in lactate (without a significant increase in the LPR) only on *A side.* Meanwhile, on *B side,* no significant differences were observed in these values. Conversely, in the SAH patients, several metabolite changes were noted. On *A side* we found a significant decrease in glucose levels, an increase in glycerol, lactate and in piryvate, while on *B side* a significant increase in glucose, glycerol and pyruvate levels was observed. These results suggest that *B side* in SAH is more prone to local metabolic disturbances than *B side* in TBI. Presumably, in TBI the brain damage is more local than in SAH pathology, and the contralateral hemisphere maintains a certain capacity to autoregulate under stress conditions. Contrarily, in SAH patients the dysregulation seems to be more global, putting both hemispheres at risk of developing secondary damage. Therefore, our findings suggest that some neurocritical patients may benefit more from bilateral monitoring than others. Arguably, in patients affected by SAH, bilateral monitoring may help for enhancing the detection of ischemic events. Furthermore, analyzing metabolites on the control side (*C side*) enabled us to confirm that the observed changes were specific to the brain tissue.

## Conclusions

Our results suggest that IHT may lead to disturbances in the brain metabolism of neurocritical ill patients, as evidenced by the changes observed in MD registries. The metabolic alterations seemed to be more pronounced on the hemisphere with more primary damage than in the contralateral one, particularly in patients with TBI. However, it is important to note that IHT may also cause some degree of impairment, albeit less severe, on the contralateral hemisphere, especially in SAH patients. Arguably, the impact of acquired brain injury on autoregulation may vary regionally depending on the specific pathology involved.

### Limitations

The main limitation of the present study, as with most studies on MD, is the small sample size. Specifically, the subgroup analyses according to the underlying etiology of brain damage included only data from 17 SAH patients with 47 IHT and 10 TBI patients with data from 20 IHT. Nonetheless, we found significant differences and new intriguing data which warrants further research on the topic. On the other hand, we defined the *A side* as the hemisphere with more damage. However, in some patients the brain damage might be more widespread or global. It would have been beneficial to subclassify patients with more generalized brain damage and those with more localized damage. However, the limited number of patients in the study precluded the evaluation of smaller subgroups of patients without compromising the statistical validity and generalizability of the findings. Further studies with larger sample sizes are needed.

## Materials and methods

The study protocol was approved by the scientific research ethics committee of Hospital Clinic of Barcelona, and informed consent was obtained from all participants and/or their legal guardians. The protection of the personal data of the participants, as well as the standards for good clinical practices, complied with what is contemplated in the Declaration of Helsinki. The identities of the participants were protected by anonymizing the data. The methodology of this report follows the recommendations of the Strengthening the Reporting of Observational Studies in Epidemiology (STROBE) guidelines.

### Study design

The study was held in a third-level stroke-referral hospital between January 2017 and January 2019. The targeted cohort comprised neurocritical patients who were eligible for multimodal neuromonitoring, particularly with microdialysis (MD). Therefore, both neurovascular patients with high-grade subarachnoid hemorrhage (SAH) (i.e. WFNS grade 4 and 5) and patients suffering from severe traumatic brain injury (TBI) (i.e. GCS ≤ 8) harboring intraparenchymal contusions were included. Data were prospectively collected, from hospital admission to three months after the initial brain insult. Supplementary Fig. S1 illustrates the study design.

Inclusion criteria were (1) admission with high-grade non-traumatic SAH or with severe TBI (2) ≥ 18 years of age, (3) neuromonitoring including bilateral MD and (4) at least one brain CT scan during the time of MD-monitoring. We excluded (1) patients in whom a MD probe was dysfunctional, (2) patients lacking head CT scans during monitoring period.

#### Neurocritical care and monitoring strategy

In our institution, neurocritical patients are admitted to a dedicated intensive care unit. Multimodal monitoring strategies are chosen by consensus between neuro-intensivists and neurosurgeons, following an internal protocol. Basic monitoring in our unit includes invasive intracranial pressure (ICP) monitoring and tissue oxygen pressure (PitO2). In cases of acute hydrocephalus, an external ventricular drain was placed as soon as possible, and was used for hourly determination of ICP. Besides, in every patient, a double sensor of ICP and PitO2 was inserted in an intraparenchymal and unilateral manner. This served for constant registration of ICP and PitO2. The sensor was placed in the white matter of the frontal lobe, about 2–4 cm anterior to the coronal suture, 2–3 cm lateral to the midline, and about 3 cm deep from the inner table of the skull. The sensor was inserted in the hemisphere considered more vulnerable to develop cytotoxic or vasogenic edema (i.e. secondary brain injury, SBI), to detect PitO2 decreases and/or ICP increases as soon as possible. In SAH cases, it was placed ipsilateral to the site of the ruptured aneurysm or ipsilateral to the intracranial hematoma (if present). In cases of unclear laterality (i.e. anterior communicating or basilar artery aneurysms), the non-dominant hemisphere was selected. In TBI cases, the sensor was placed in the hemisphere with a higher volume of hemorrhagic damage or hemispherical swelling. In diffuse injuries corresponding to Marshall type I or III, it was placed in the non-dominant hemisphere.

In high-grade SAH and severe TBI, an additional monitoring with bilateral brain microadialysis (MD) probes to follow metabolic changes due to SBI is considered. The first probe is introduced in the hemisphere at higher risk of SBI, as previously explained. To the aims of this study, this hemisphere has been termed *side A*. In SAH cases, the probe target is the white matter, within the watershed area between the anterior cerebral and middle cerebral artery vascular territories. In TBI cases, first probe is places within the area of the so-called “traumatic penumbra”, i.e. the potentially salvageable brain tissue surrounding the primary lesion. A contralateral probe was located in the other hemisphere, herewith defined as *B side.* Additionally, a subcutaneous probe was placed in the subcutaneous fatty tissue of the abdomen. This probe was connected to the infusion pump, just like the brain probes, and served as a control for systemic changes in the metabolites.

According to the institutional protocol, MD probe was implanted within the first 24 h of ICU admission. If emergent surgical or endovascular treatment are deemed, the MD probes are inserted afterwards. Technically, craniometric points are used to locate the selected entry point. A manual 5.8 mm twist drill is used to make a bilateral burr hole, the dura is opened, and a blunt needle is used to create the intraparenchymal trajectory. The probe is inserted up to the preplanned depth. The distal end is tunneled 5 cm under the skin and fixed with silk suture. The catheter probe is connected to the pump and the infusion is initiated. A computed tomography (CT) scan is performed within the first 24 h of MD probe insertion to rule out complications and to verify it is within the desired position. If malposition or incidental pull-out are detected, the probe is relocated as soon as possible (and always within one hour). MD samples are collected by trained nurses hourly.

#### In hospital transfers

In our institution patients admitted to the ICU requiring neuroimaging (CT or MR) or invasive neurosurgical or endovascular procedures need to be transferred either to the radiology department or to the operating room or angio-suite. These are in the same building as the ICU, but not on the same floor, making it necessary to use the elevator during transfer. In addition, it is necessary to change the patient’s stretcher to carry out the transfer, both on the way to and from the CT or the operating room. During the IHT, patients were accompanied by a specialized neuro-intensivist and nursing staff from the same unit. The external ventricular drain was closed, and the ICP and PtiO2 monitoring disconnected, but the MD perfusion pump remained active throughout the transfer.

In this study, all IHT were recorded. For the analysis, IHT carried out for routine neuroimaging and surgical or endovascular treatments were considered. Conversely, IHT performed on unstable patients, patients with increased ICP or with pupillary changes were excluded. Likewise, patients who developed an acute worsening 24 h after IHT or those who did not return to the ICU after performing the imaging because surgery was needed were also excluded. The duration of each IHT was registered prospectively. Technical complications recorded during transfer were collected retrospectively.

#### Sedation protocol

To maintain patient sedation during transfer, continuous propofol and remifentanil were infused (sedation measured by the Bispectral Index (BIS) in the range of 40 to 60). In addition, consistent ventilation was controlled by Dräger Oxylog 3000 transport respirator device (Lübeck, Germany).

#### Vasospasm detection and management in high-grade SAH

Patients with high-grade SAH who cannot be clinically evaluated (under sedation or comatose), are regularly screened for vasospasm, between day 2 and day 21 after the bleeding. A transcranial doppler (TCD) ultrasound is performed twice a day by dedicated intensivists to detect TCD vasospasm, which was defined as a mean flow velocity in any vessel > 120 cm/sec^19^. If increased mean flow velocities are detected, a multimodal CT (basal CT, angio-CT and CT perfusion) is performed. Besides, a multimodal CT is performed every two days in every patient, starting from day + 3 and up to day + 14 of the hemorrhage.

Angiographic vasospasm is a condition that occurs after a ruptured brain aneurysm, where the blood vessels in the brain constrict due to components released from the bleeding in the subarachnoid space. This constriction can lead to decreased blood flow and potentially cause further damage to the brain^20^. Angiographic vasospasm, which can be detected by using DSA or CTA scanning, is defined as a significant arterial narrowing > 20%. Meanwhile, delayed cerebral ischemia (DCI) is defined as neurological deterioration of ≥ 2 points in GCS or NIHSS score, lasting at least 1 h and without other plausible causes.

When vasospasm is detected in TCD or multimodal CT, medical management is started, by means of hypertension and hypervolemia, with a targeted mean arterial pressure of 110–120 mmHg. If medical management fails to improve the delays in cerebral perfusion maps, endovascular management is sought. First line endovascular therapy includes intraarterial local infusion of verapamil. Second line therapy consists of balloon angioplasty.

### Clinical data compilation

The following clinical data were prospectively collected: sex, age, date and number of IHT per patient, surgical and/or endovascular treatments, and development of DCI. In SAH patients, radiological vasospasm was defined as the presence of a new-onset narrowing of a vessel, documented in either computed tomography angiography (CTA) or digital-subtraction angiography (DSA)^21^.

Upon admission to the emergency department, the neurological state was recorded according to GCS. For TBI patients, GCS 3–8 was considered as severe; 9–12 as moderate; and 13–15 as mild. In SAH patients, WFNS IV-V was considered as severe; II-III as moderate; and I as mild. Upon discharge, patients were evaluated for midterm follow-up on day 90 after the SAH or TBI onset. This final evaluation included a neurological examination and a functional evaluation. Functional outcome was assessed with the modified Rankin Scale (mRS) or with the Glasgow Outcome Scale-Extended (GOSE) at 3 months through an in-person visit following structured questionnaires. A mRS of 0–2 was considered a good clinical outcome in SAH patients. A GOSE of 4 (moderate disability with some independence) or 5 (good recovery) was considered a good outcome for TBI patients, while 1 (death), 2 (vegetative state), or 3 (severe disability requiring daily care) were considered as poor outcome.

### Bilateral MD monitoring data

**Figure 4:**
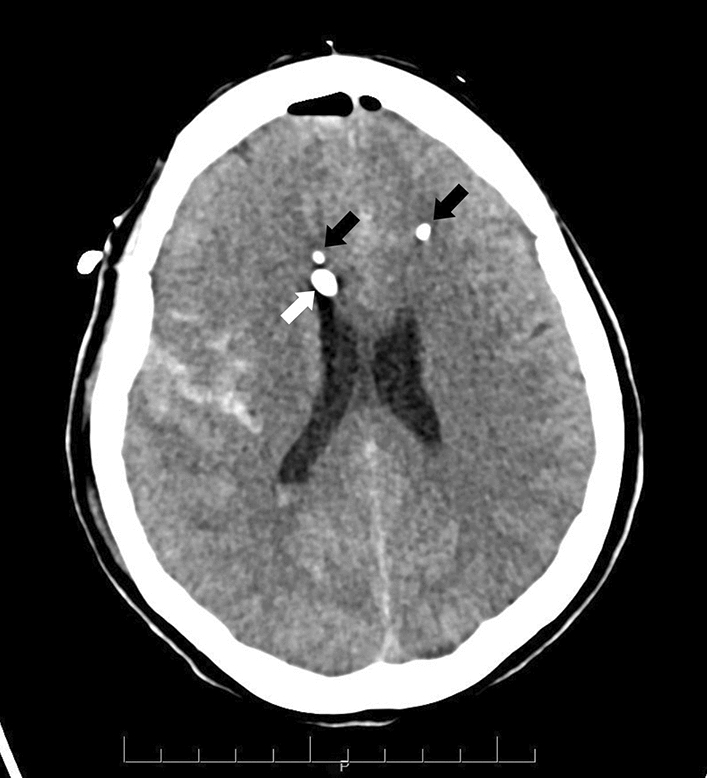
Axial cut of a cerebral CT scan of a patient with SAH. The figure shows the bilateral placement of the microdialysis probes (black arrows) and the external ventricular drain (white arrows). The CT scan has been anonymized.

MD was measured using a 20 kDa catheter (CMA 70; CMA/Microdialysis, Solna, Sweden) with a membrane length of 10 mm. As mentioned before three catheters were inserted, two into the brain (Fig. 4) and the other one in subcutaneous. The catheter was inserted intraparenchymal and bilaterally, and connected to a perfusion pump (CMA 106; CMA/Microdialysis). Catheters were classified as *A side* (highest risk of SBI) and *B side* (contralateral), as previously defined^7,8^.

For MD monitoring, the perfusion fluid (CMA mosm/Kg) was infused at a flow rate of 0.3 µl/minute. The microvials were collected and interpreted every hour, without interrupting the collection during transfer or procedures performed on the patient. Lactate, pyruvate, glycerol and glucose concentrations were analyzed by conventional microdialysis ISCUS FLEX H107263 analysis equipment (CMA600; CAM/Microdialysis). Bilateral MD data were collected 10 h before transfer, during the transfer, and 10 h after transfer, thus collecting a total of 21 h of MD monitoring per patient and per transfer. Cases with < 80% of samples collected during the 21 h were excluded. Data recording was stopped if one or both catheters consistently reported errors (5 ± 2 days).

All monitored patients underwent a safety protocol. The safety protocol included: (1) Daily revision of catheter wounds to identify CSF leak or infection; (2) once the monitoring was stopped, the catheter tip was sent to the lab to detect microorganisms; (3) a CT was routinely performed after catheter placement in order to detect hemorrhagic complications related to the surgery and to confirm the location of the catheter tip.

### Statistical analysis

Statistical analyses were performed with SPSS v.25.0 and with Graph Prism v.5.0. For categorical or nominal values, a chi-square test was performed and for numerical values, a *t* test was performed to compare the means of two groups. Before performing the test, the normal distribution of numerical values was evaluated with the Shapiro test to determine whether the normal distribution could be assumed. Only the levels of glucose in *A side* and the levels of glucose, lactate and pyruvate in *C side*, passed the normality test. In normal distributed variables, the two-tailed paired t-test was applied. In those cases where the distribution was not normal, a non-parametric two-tailed Wilcoxon test was applied. The level of significance was established at a 0.05 level (2-sided). The graphs were obtained with Graph Prism v.5.0.

### Supplementary Information

Supplementary Information.

## Data Availability

This study was registered after the study began. The study is registered at OSF with the number: osf.io/snmy5. The analysis plan was not formally pre-registered, but the team member with primary responsibility for the analysis (lead author, RT) certifies that the analysis plan was pre-specified. Data from this study are available in a public archive: osf.io/snmy5. There is no analytic code associated with this study.
