# Supplementary material for: Brain metabolism response to intrahospital transfers in neurocritical ill patients and the impact of microdialysis probe location
Source: Sci Rep. 2024 Mar 28;14:7388. doi: 10.1038/s41598-024-57217-5 (PMC10978944; doi:10.1038/s41598-024-57217-5)
Supplement: Supplementary file 1 — Supplementary Information. [file 41598_2024_57217_MOESM1_ESM.docx]

**Brain metabolism response to intrahospital transfers in neurocritical ill patients: Does the microdialysis probe location matter?**

Leire Pedrosa^1,2^, Jhon Hoyos^1^, Luis Reyes^1^, Alejandra Mosteiro^1^, Luigi Zattera^3^, Thomaz Topczewski^1^, Ana Rodríguez-Hernández^4^, Sergio Amaro^2,5^, Ramon Torné*^1,2,5^, Joaquim Enseñat^1^.

| **TBI (n=10)** | | |
| --- | --- | --- |
|  |  | **n (%)** |
| **Injury side** | **Bilateral frontal contusions** | 2 (20%) |
|  | **Right acute subdural hematoma** | 5 (50%) |
|  | **Left temporal contusion and subdural hematoma** | 3 (30%) |
| **Surgery side** | **Right** | 3 (100%) |
|  | **Left** | 0 (0%) |

**Supplementary Table S1: Injury- and surgery side of TBI patients**

| **SAH (n=17)** | | |
| --- | --- | --- |
|  |  | **n (%)** |
| **Aneurism location** | **MCA Right** | 5 (29%) |
|  | **MCA Left** | 1 (6%) |
|  | **ACoA** | 4 (24%) |
|  | **ACoP** | 3 (18%) |
|  | **AICA** | 1 (6%) |
|  | **ACoA + ACA Left** | 1 (6%) |
|  | **ACA Left** | 1 (6%) |
|  | **PICA Left** | 1 (6%) |
| **Surgery side** | **Right** | 4 (24%) |
|  | **Left** | 3 (18%) |

**Supplementary Table S2: Aneurism location and surgery side of SAH patients**

|  |  | **A side** | | | **B side** | | |
| --- | --- | --- | --- | --- | --- | --- | --- |
| **Metabolite** | **Pathology** | **Pre IHT** | **Post IHT** | **p-value** | **Pre IHT** | **Post IHT** | **p-value** |
| **Glucose (mmol/L) ± SD** | **SAH-non DCI** | 1,975 ± 0,9858 | 1,770 ± 0,9474 | **0.009** | 1,956 ± 1,646 | 1,620 ± 1,057 | **0.018** |
| **Glycerol (Umol/L) ± SD** | **SAH-non DCI** | 331,0 ± 308,3 | 377,3 ± 321,3 | **0.035** | 232,4 ± 173,0 | 272,3 ± 174,2 | **0.013** |
| **Lactate (mmol/L) ± SD** | **SAH-non DCI** | 3,964 ± 2,277 | 4,912 ± 2,890 | **<0,0001** | 3,726 ± 2,535 | 3,790 ±2,224 | 0.173 |
| **Pyruvate (Umol/L) ± SD** | **SAH-non DCI** | 116,8 ± 54,51 | 133,7 ± 53,00 | **0.0006** | 110,3 ± 55,10 | 117,0 ± 47,46 | **0.058** |
| **LPR ± SD** | **SAH-non DCI** | 39,56 ± 35,91 | 46,95 ± 60,76 | 0.168 | 33,31 ± 17,91 | 31,80 ± 14,48 | 0.557 |

**Supplementary Table S3:** **Metabolites analyzed by MD in both hemispheres (A and B side) pre- and post-IHT in non-DCI SAH patients.**


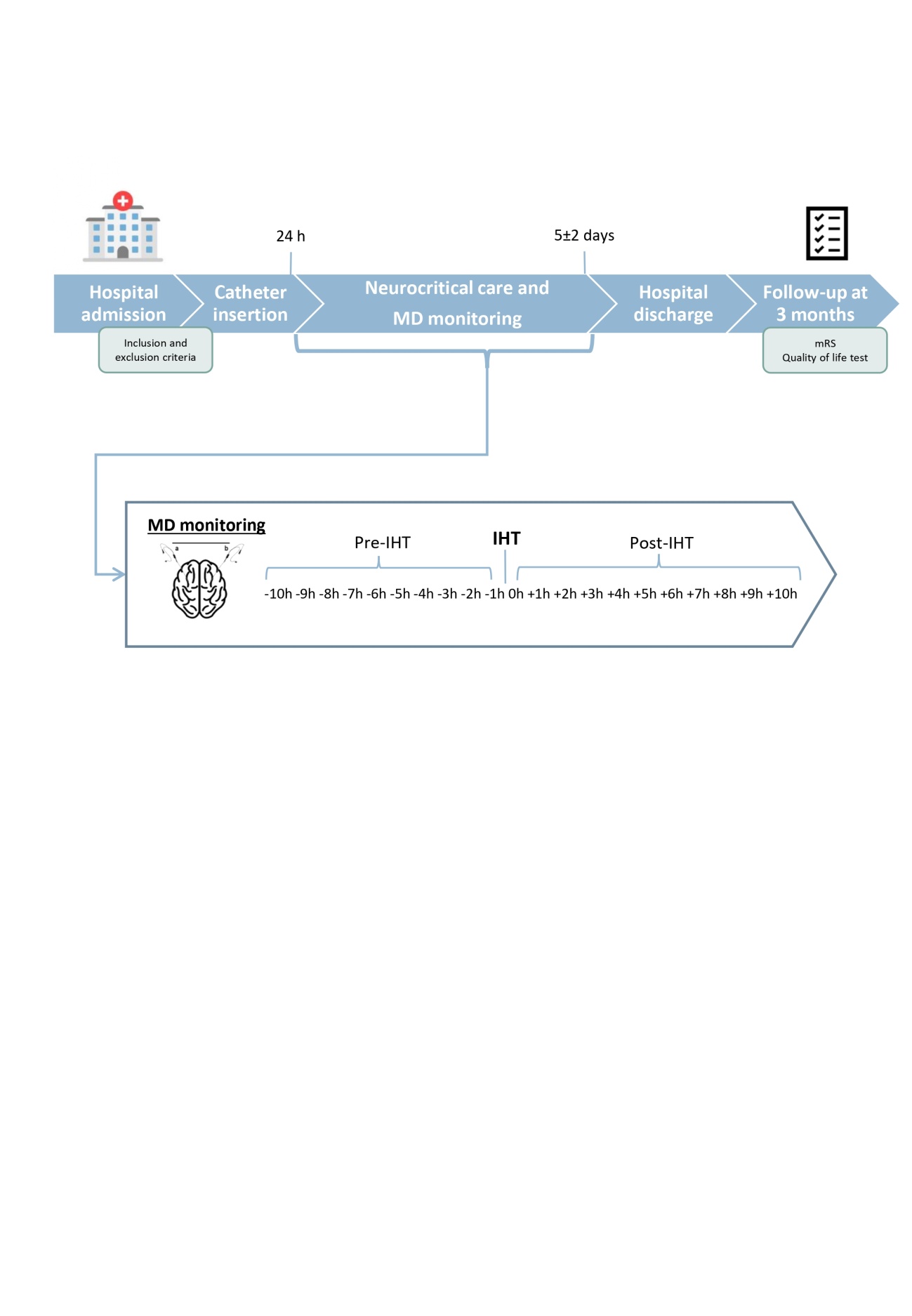


**Supplementary Figure S1: Schema of study design.**
